# Evaluation of Xpert GBS assay and Xpert GBS LB assay for detection of *Streptococcus agalactiae*

**DOI:** 10.1186/s12941-021-00461-8

**Published:** 2021-09-06

**Authors:** Meng-Yi Han, Chen Xie, Qing-Qing Huang, Qiao-Hua Wu, Qing-Yun Deng, Tian-Ao Xie, Ye-Ling Liu, Zhuo-Lei Li, Jing-Hua Zhong, Yan-Chao Wang, Xu-Guang Guo

**Affiliations:** 1 Department of Clinical Laboratory Medicine, The Third Affiliated Hospital of Guangzhou Medical University, Guangzhou, 510150 China; 2 Department of Clinical Medicine, The Third Clinical School of Guangzhou Medical University, Guangzhou, 511436 China; 3 Key Laboratory for Major Obstetric Diseases of Guangdong Province, The Third Affiliated Hospital of Guangzhou Medical University, Guangzhou, 510150 China; 4 Key Laboratory of Reproduction and Genetics of Guangdong Higher Education Institutes, The Third Affiliated Hospital of Guangzhou Medical University, Guangzhou, 510150 China; 5 Experimental Department of Institute of Obstetrics and Gynecology, The Third Affiliated Hospital of Guangzhou Medical University, Guangzhou, 510150 China; 6 Department of Clinical Medicine, The Second Clinical School of Guangzhou Medical University, Guangzhou, 511436 China

**Keywords:** Xpert GBS assay, Xpert GBS LB assay, Group B *Streptococcus*, Diagnostic accuracy

## Abstract

**Background:**

Group B *Streptococcal* (GBS) infection is the primary agent of neonatal morbidity and mortality. Rapid and simple methods to detect GBS are Xpert GBS and GBS LB assays based on real-time polymerase chain reaction (PCR). However, since the diagnostic accuracy of the two techniques in diagnosing GBS remains unclear, we designed this study to appraise the diagnostic accuracy of the aforementioned.

**Methods:**

A systematic search of all literature published before July 16, 2020 was conducted using Embase, PubMed, Web of Science, and Cochrane Library. The study quality was evaluated through Review Manager 5.3. Accordingly, data extracted in the included studies were analyzed using Meta-DiSc 1.4 and Stata 12.0 software. The diagnosis odds ratio (DOR) and bivariate boxplot were utilized to evaluate the heterogeneity. Publication bias was appraised by using Deeks’ funnel plot.

**Results:**

A total of 13 studies were adopted and only 19 sets of data met the criteria. The sensitivity and specificity of Xpert GBS were 0.91 (95% CI 0.89–0.92) and 0.93 (95% CI 0.92–0.94). The area under the curve (AUC) was 0.9806. The sensitivity and specificity results of Xpert GBS LB were 0.96 (95% CI 0.95–0.98) and 0.94 (95% CI 0.92–0.95), respectively. The AUC was 0.9950. No publication bias was found.

**Conclusions:**

The Xpert GBS and GBS LB assays are valuable alternative methods with high sensitivity and specificity. However, determining whether they can be used as clinical diagnostic standards for GBS is essential for the future.

**Supplementary Information:**

The online version contains supplementary material available at 10.1186/s12941-021-00461-8.

## Background

*Streptococcus agalactiae*, also known as group B *Streptococcus* (GBS), is a gram-positive *Streptococcus* inhabiting the vagina and rectum of humans [1]. GBS may reproduce briefly, intermittently, or continuously on the vaginal or anorectal mucosa of a woman [2]. Worldwide, the estimated incidence of systemic invasive GBS in pregnant women is 0.38 cases per 1000, with a case fatality rate of 0.2% [3]. China has an estimated 13,604 cases of GBS and 1141 deaths of GBS-related infants aged less than 90 days each year [4]. GBS can be vertically transmitted from a pregnant woman to a newborn at birth, causing early (day 6) or late (day 7 to day 89) disease, a common cause of neonatal infection and death [5, 6].

The American College of Obstetricians and Gynecologists (ACOG) guidelines in 2019 recommend GBS screening for pregnant women with a gestational age between 36 0/7 and 37 6/7 weeks and intrapartum antibiotic prophylaxis (IAP) treatment for women with positive results. Currently, enrichment culture is the gold-standard method for detecting GBS [7–9]. However, with limitations: (a) it requires 18–72 h; (b) the source of specimen collection and the experience of laboratory operators have a significant impact on its accuracy; (c) sensitivity is estimated at 54.3 to 83.3%. In addition, as enrichment culture takes time to obtain results, some pregnant women who don't receive prenatal care before presenting in labor and those who give birth prematurely, before the recommended testing period cannot be helped [10, 11]. Moreover, the 2010 CDC revision of the guidelines mentioned that nucleic acid amplification tests (NAAT) such as polymerase chain reaction (PCR) could be used to assess intrapartum GBS colonization with the advantage of rapid turnaround time. But the sensitivity of the direct-from-specimen testing is not adequate compared to culture [1]. Futhermore, traditional PCR requires technically skilled operators and specific laboratory equipment, thereby limiting its practical use [12].

Presently, increasing real-time PCR assays for GBS have been developed, such as Xpert GBS (Cepheid, USA) and Xpert GBS LB (Cepheid, USA). Cepheid’s Gene Xpert System can automatically extract, amplify, and detect DNA, a system that could be installed out of the laboratory in countries approved by regulatory bodies, only requiring simple operation and providing results in less than 1 h, resulting in a more practical method for the rapid diagnosis of GBS [11, 13]. It targets the CAMP factor encoding gene present in nearly all GBS [14]. Furthermore, Xpert GBS and Xpert GBS LB are both based on the Gene Xpert System for the antepartum and intrapartum screening of GBS. They differ fundamentally in that the specimen of Xpert GBS LB requires enrichment before detection, while Xpert GBS is performed from the primary specimen without enrichment [15]. However, due to the lack of systematic evaluation of the accuracy of Xpert GBS and Xpert GBS LB in detecting GBS, we conducted this research to assess the sensitivity and specificity of these methods to provide a new method for clinical rapid diagnosis of GBS.

## Methods

### Study design

Studies published before July 16, 2020 were considered for the literature review. The accuracy of the Xpert GBS and GBS LB diagnostics in the GBS was systematically reviewed through a pooled-analysis.

### Search strategy

We searched all literature dated before July 16, 2020 in four major databases—PubMed, Embase, Web of Science, and Cochrane Library—using the following search strategy: ((“Xpert GBS” OR “Xpert GBS technology” OR “Xpert GBS assay” OR “Xpert® GBS real-time PCR” OR “Xpert GBS real-time PCR assay” OR “Xpert GBS real-time PCR kit” OR “Xpert GBS rapid test”) AND “Group B Streptococcal [all synonyms]”).

### Study selection

Inclusion criteria: Studies were included if (1) Xpert GBS or GBS LB assay was used as a detection method; (2) complete data were extractable to construct fourfold tables; (3) the reference standard was described, including GBS culture (enrichment culture or direct culture) or PCR; (4) they were published in English; (5) the sample size was no fewer than 50.

Exclusion criteria: Studies were excluded when (1) duplicate studies existed; (2) there was a lack of complete data for fourfold tables; (3) no reference standard or composite reference standard, including Xpert GBS or GBS LB assay, were provided; (4) abstracts or conference summary.

### Data extraction and quality assessment

All our data were extracted independently by four experimenters, followed by checking and verification by other experimenters. Any existing differences were verified by other experimenters twice until correct.

The Quality Assessment of Diagnostic Accuracy Studies (QUADAS-2) guideline was used to evaluate the quality of the included study that has four key parts: patient selection, index test, reference standard, and flow and timing [16]. The quality of studies was plotted using Review Manager version 5.3.

### Statistical analysis

Meta-DiSc version 1.4 was employed to estimate the extracted fourfold table data: sensitivity, specificity, positive likelihood ratio (PLR), negative likelihood ratio (NLR), diagnostic odds ratio (DOR), together with 95% confidence intervals (95% CIs). Subsequently, a summary receiver operating characteristic (SROC) curve was plotted based on pooled sensitivity and specificity, followed by conducting a random effects model to analyze the data; the results were presented via forest maps. We used Stata version 12.0 to draw Deeks’ funnel plot to detect publication bias along with generating a bivariate box plot and Fagan’s nomogram to evaluate the outliers and describe the diagnosis value of Xpert GBS and GBS LB assays for GBS.

In addition, we found that if the true positive (TP) or true negative (TN) of the four-fold table data was 0, errors would occur in data analysis. In order to solve this problem, we changed 0 to 0.5 in meta-DISc and 0 to 1 for Fagan’s nomogram in Stata.

## Results

### Eligible studies

We searched 45 related studies from four databases (19 in PubMed, 4 in Embase, 22 in Web of Science, and 0 in Cochrane Library) according to the search strategy, excluding 23 duplicates of these. Moreover, none of the studies were excluded after reviewing the abstract. Therefore, by screening the full text of the 22 studies, 9 were excluded due to various reasons (2 studies lacked gold standard, 2 studies were short of enough samples, 1 had composite gold standard including detection method, 1 was a conference summary, and 3 had incomplete data). At last, we incorporated 13 studies meeting the inclusion and exclusion criteria [2, 10–15, 17–22]. More details have been provided in the supplementary materials (Additional file 1: Figure S1).

### Study result & characteristics

In the selected 13 studies, we obtained 19 groups of data and 6273 samples. From these studies, we identified information such as author, year, country, reference standard, detection method, sample type, sample source, the timing of specimen collection and pregnancy time. The detailed characteristics of the included studies are summarized in Table 1.

**Table 1:** Characteristics of included studies

Author	Year	Country	Study design	Reference standard	Detection method	Sample type	Sample source	The timing of specimen collection	Pregnancy time	The number of sample	TP	FP	FN	TN
El Helali	2009	French	Prospective	Culture^a^	Xpert GBS	Vaginal	Hospital	Intrapartum	≥ 35 weeks	863	135	3	2	723
Bourgeois-Nicolaos	2013	French	Prospective	Direct culture^b^	Xpert GBS	Amniotic fluid	Hospital	Intrapartum	≥ 37 weeks	139	10	2	1	126
Park	2013	South Korea	Prospective	Enrichment culture^c^	Xpert GBS	Vaginal-rectal	Hospital	Antepartum	35–39 weeks	175	13	7	2	153
Buchan	2014	America	Prospective	Enrichment culture^c^	Xpert GBS LB	Vaginal-rectal	Hospital	Antepartum	35–37 weeks	826	189	48	2	587
Buchan	2014	America	Prospective	Enrichment culture^c^	Xpert GBS	Vaginal-rectal	Hospital	Antepartum	35–37 weeks	505	96	15	16	378
Gouve	2016	Brazil	Prospective	Enrichment culture^c^	Xpert GBS	Vaginal-rectal	Hospital	Antepartum	35–37 weeks	336	55	14	9	258
Helmig	2017	Denmark	Prospective	Enrichment culture^c^	Xpert GBS	Vaginal-rectal	Hospital	Intrapartum	≥ 34 weeks	105	25	2	0	78
Rabaan	2017	Saudi Arabia	Prospective	Enrichment culture^c^	Xpert GBS	Vaginal-rectal	Clinics	Antepartum	35–37 weeks	554	139	44	0	371
Plainvert	2017	France	Prospective	Direct culture^b^	Xpert GBS	Vaginal	Hospitals	Intrapartum	> 34 weeks	516	238	56	44	178
Said	2018	South Africa	Prospective	Direct culture^b^	Xpert GBS	Vaginal-rectal	Hospitals	Antepartum	26 and 37 weeks	279	61	5	9	204
Shin	2019	America	Prospective	Enrichment culture^c^	Xpert GBS LB	Vaginal-rectal	Hospitals and clinics	Antepartum	35–37 weeks	500	107	40	1	352
Vieira	2019	Brazil	Prospective	Direct culture^b^	Xpert GBS	Vaginal-rectal	Hospitals	Intrapartum	≥ 24 weeks	220	21	45	13	141
Tickler	2019	America	Prospective	Enrichment culture^c^	Xpert GBS LB	Vaginal or Vaginal/rectal	Hospitals	Unclear	Unclear	145	40	0	4	101
Tickler	2019	America	Prospective	Enrichment culture^c^	Xpert GBS LB	Vaginal or Vaginal/rectal	Hospitals	Unclear	Unclear	210	194	0	16	0
Tickler	2019	America	Prospective	Enrichment culture^c^	Xpert GBS	Vaginal-rectal	Hospitals	Unclear	Unclear	100	0	0	0	100
Tickler	2019	America	Prospective	Enrichment culture^c^	Xpert GBS LB	Vaginal-rectal	Hospitals	Unclear	Unclear	100	0	0	0	100
Tickler	2019	America	Prospective	Enrichment culture^c^	Xpert GBS LB	Vaginal-rectal	Hospitals	Unclear	Unclear	300	54	17	0	229
Tickler	2019	America	Retrospective	Enrichment culture^c^	Xpert GBS	Vaginal-rectal	Hospitals	Unclear	Unclear	150	149	0	1	0
Choera	2020	America	Prospective	Enrichment culture^c^&Revogene GBS LB assay	Xpert GBS LB	Vaginal-rectal	Hospitals	Intrapartum	35–37 weeks	250	50	1	1	198

^a^In this research, it refers to that specimens were cultured directly on blood agar plate and were subsequently placed in a broth medium

^b^In this research, it refers to that specimens were cultured directly on blood agar plate without enrichment

^c^In this research, it refers to that specimens were incubated to enrichment broth before detection

### Study quality assessment

**Fig. 1:**
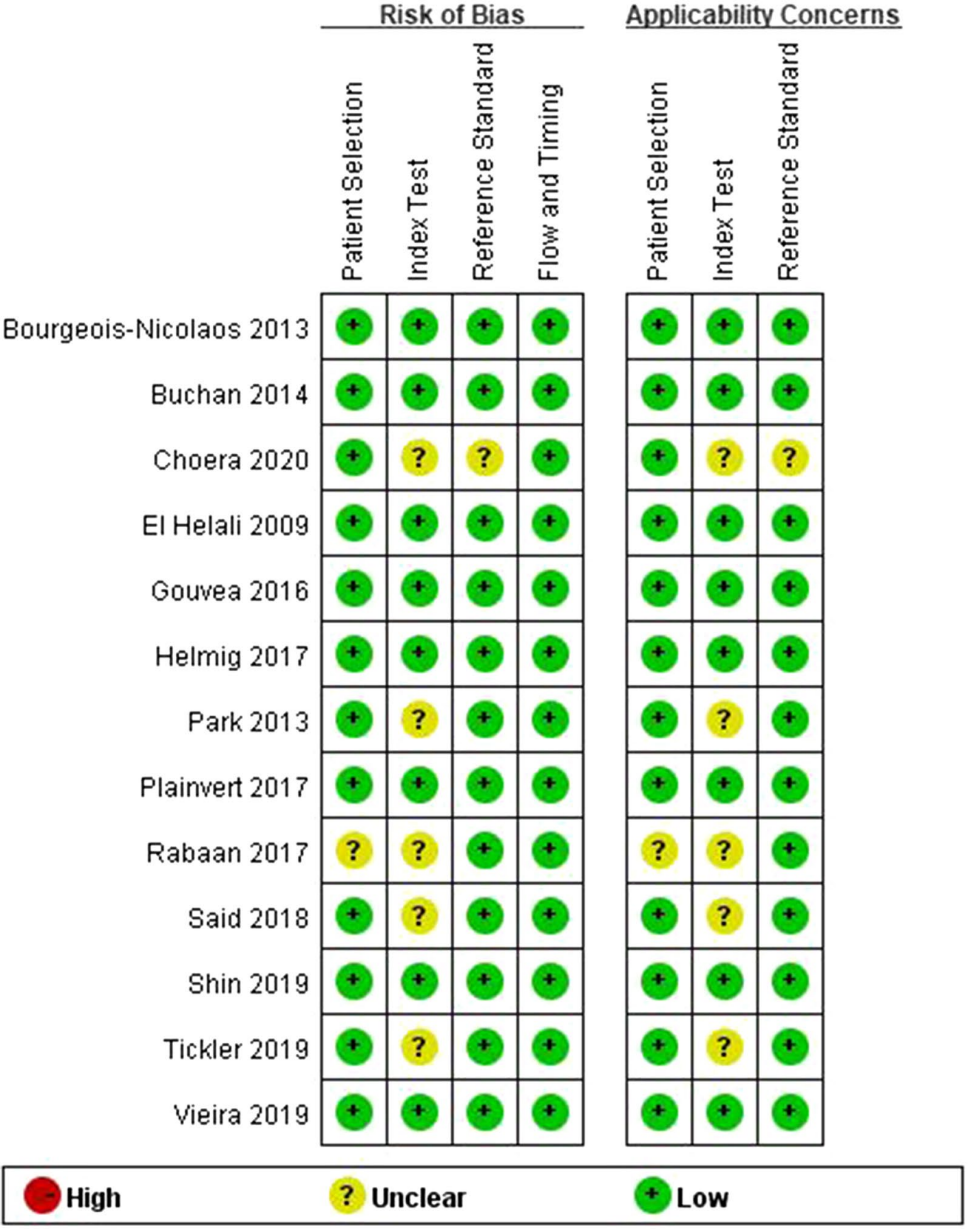
Methodological quality assessment of included studies

The quality of the individual studies was appraised by QUADAS-2 items, as shown in Fig. 1. In terms of patient selection, the results indicated that 1 study was likely to have an unclear risk of bias while the other 12 studies were at low risk. The applicability concerns of 1 study were rated as unknown and 12 as low. The index test presented that 5 studies had an unclear risk of bias when the other 8 studies had a low risk. The applicability concerns included 5 studies of unknown concerns and 8 of low concerns. In terms of reference standard, 1 study was rated an unclear risk and its applicability concern was unknown. The other 12 studies showed low risks of bias and low concerns. In terms of flow and timing, all the bias risks of included studies were rated low.

### Results of Xpert GBS

#### SROC curve and diagnostic accuracy

**Fig. 2:**
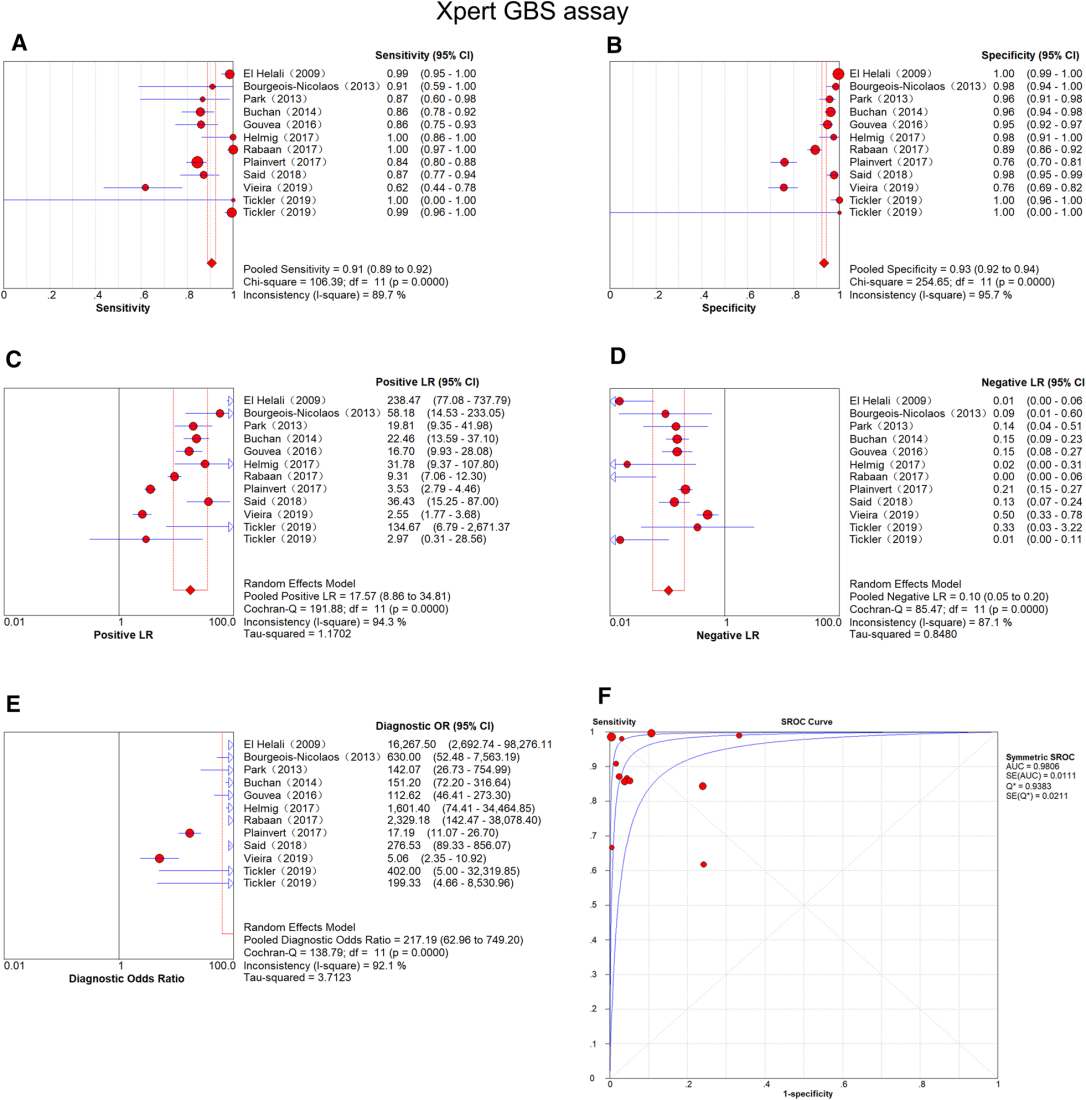
Forest plot of Xpert GBS assay. (**a** sensitivity, **b** specificity, **c** positive LR, **d** negative LR, **e** diagnostic odds ratio, **f** SROC curve)

**Fig. 3:**
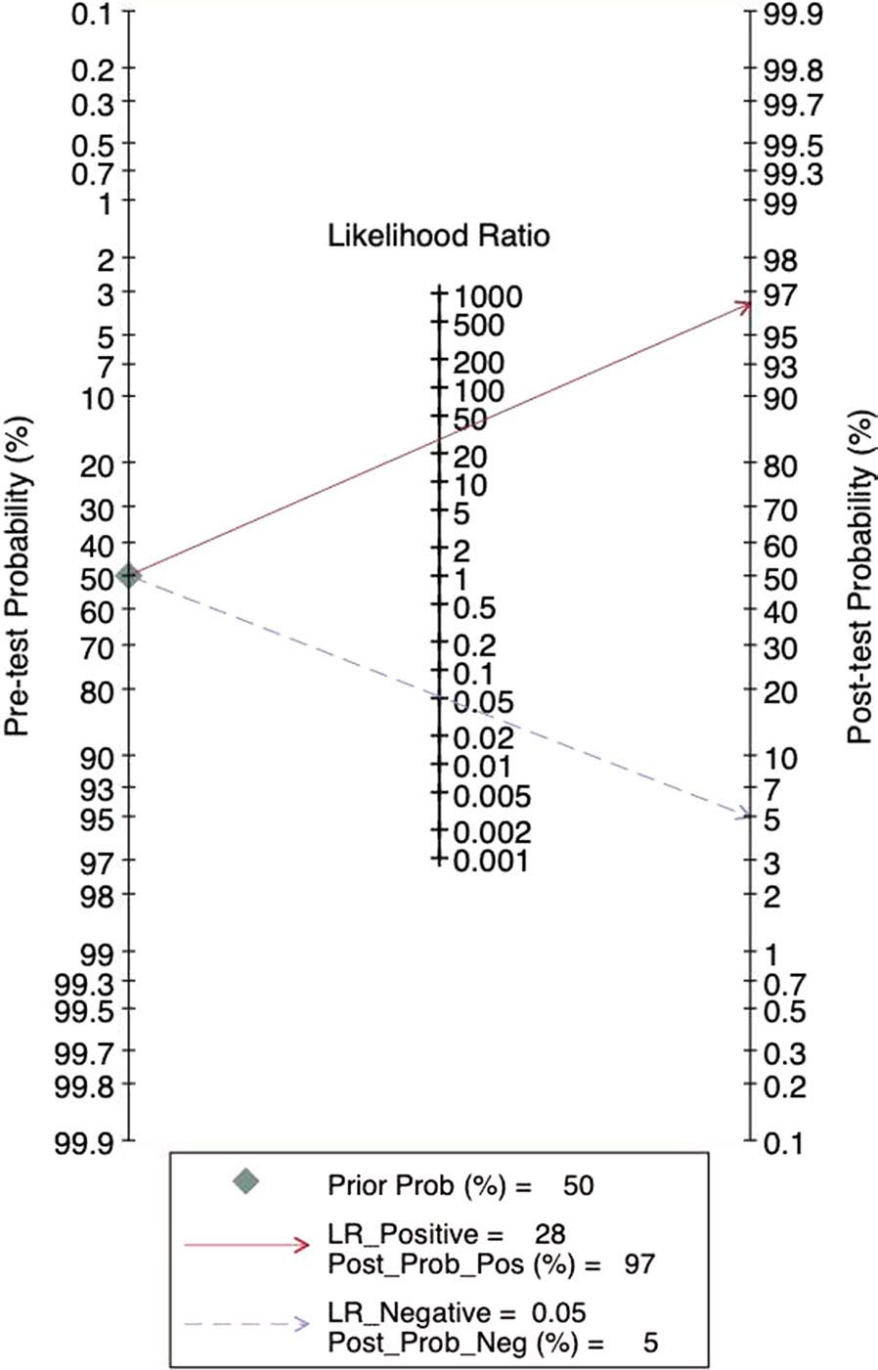
Fagan nomogram analysis of Xpert GBS assay

The results of sensitivity and specificity were 0.91 (95% CI 0.89–0.92) and 0.93 (95% CI 0.92–0.94), as shown in Fig. 2a and b, respectively. The PLR of Xpert GBS was 17.57 (95% CI 8.86–34.81) (Fig. 2c) and the NLR was 0.10 (95% CI 0.05–0.20) (Fig. 2d). The result of DOR was 217.19 (95% CI 62.96–749.20) (Fig. 2e). The AUC (the area under the SROC curve) of Xpert GBS (Fig. 2f) was 0.9806 and the Q* index was 0.9383. The Fagan nomogram analysis showed positive post-test probability (97%) and negative post-test probability (5%) when the predicted probability was set to 50% (Fig. 3).

#### Heterogeneity analysis

**Fig. 4:**
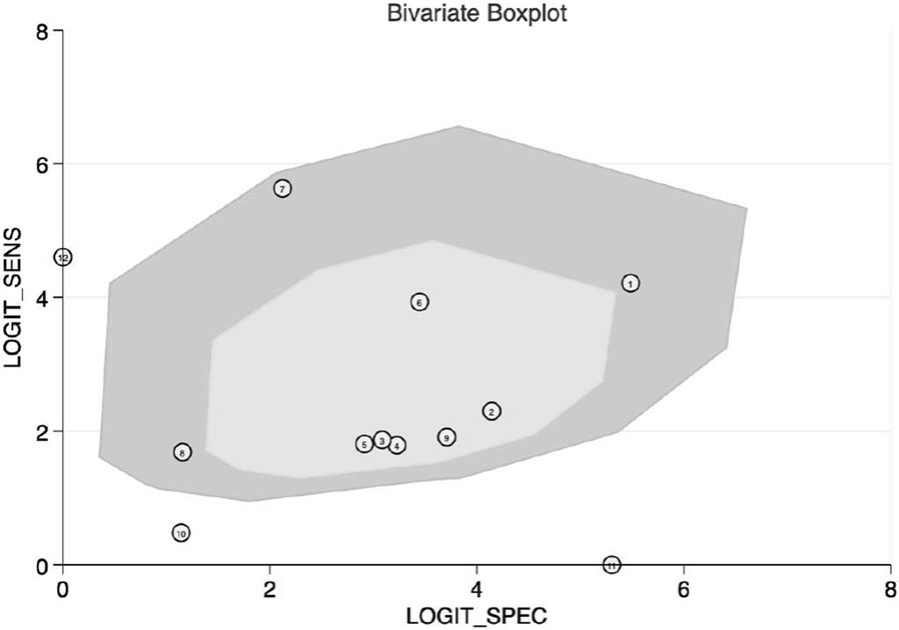
Bivariate boxplot of Xpert GBS assay

We applied the Bivariate boxplot and index (I-square) to analyze the heterogeneity. For Xpert GBS, the bivariate boxplot of Xpert GBS (Fig. 4) showed that three sets of data were out of the circles. Besides, the I^2^ of DOR was 92.1% (Fig. 2e). We used the Spearman correlation coefficient to evaluate the threshold effect of the included studies. With the studies that used Xpert GBS, the Spearman correlation coefficient was −0.063 and their *p* value was 0.846.

#### Publications bias evaluation

**Fig. 5:**
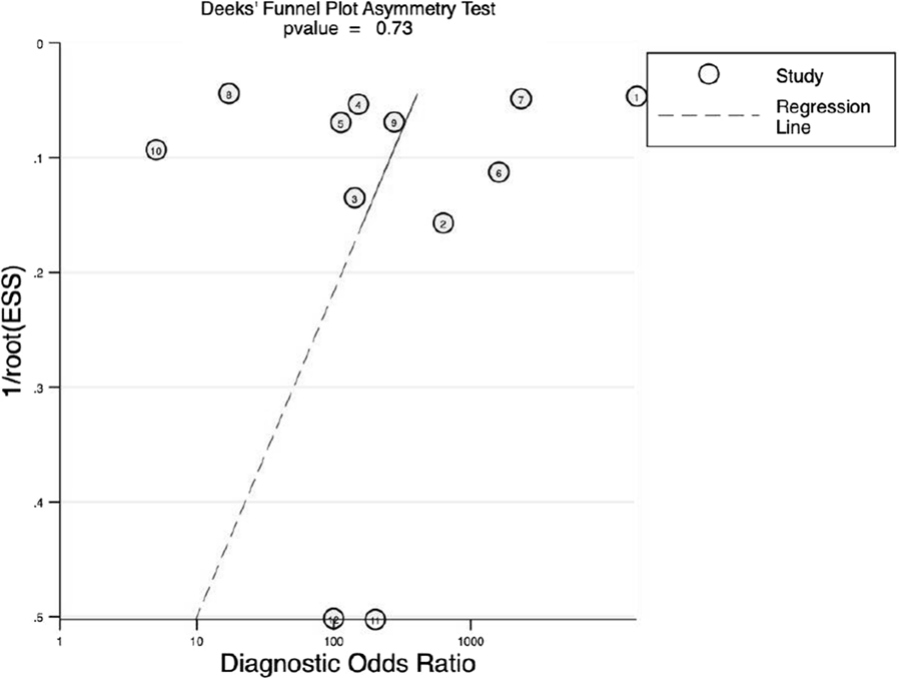
Deeks’ funnel plot asymmetry test of Xpert GBS assay

We used Deeks’ funnel plot to evaluate the publication bias [23]. Xpert GBS assay is shown in Fig. 5. The *p* value of Xpert GBS is 0.73.

### Results of Xpert GBS LB

#### SROC curve and diagnostic accuracy

**Fig. 6:**
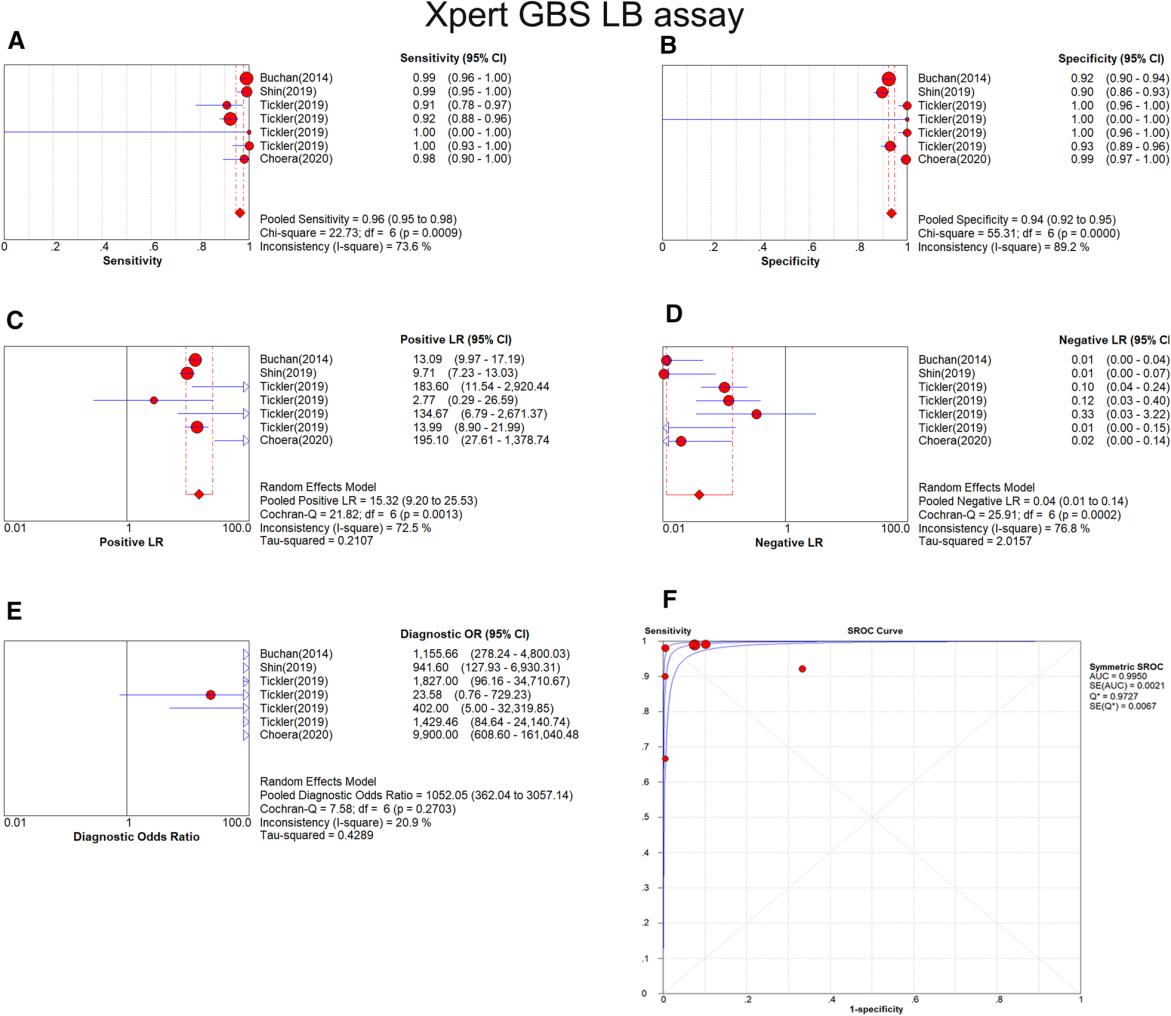
Forest plot of Xpert GBS LB assay. (**a** sensitivity, **b** specificity, **c** positive LR, **d** negative LR, **e** diagnostic odds ratio, **f** SROC curve)

**Fig. 7:**
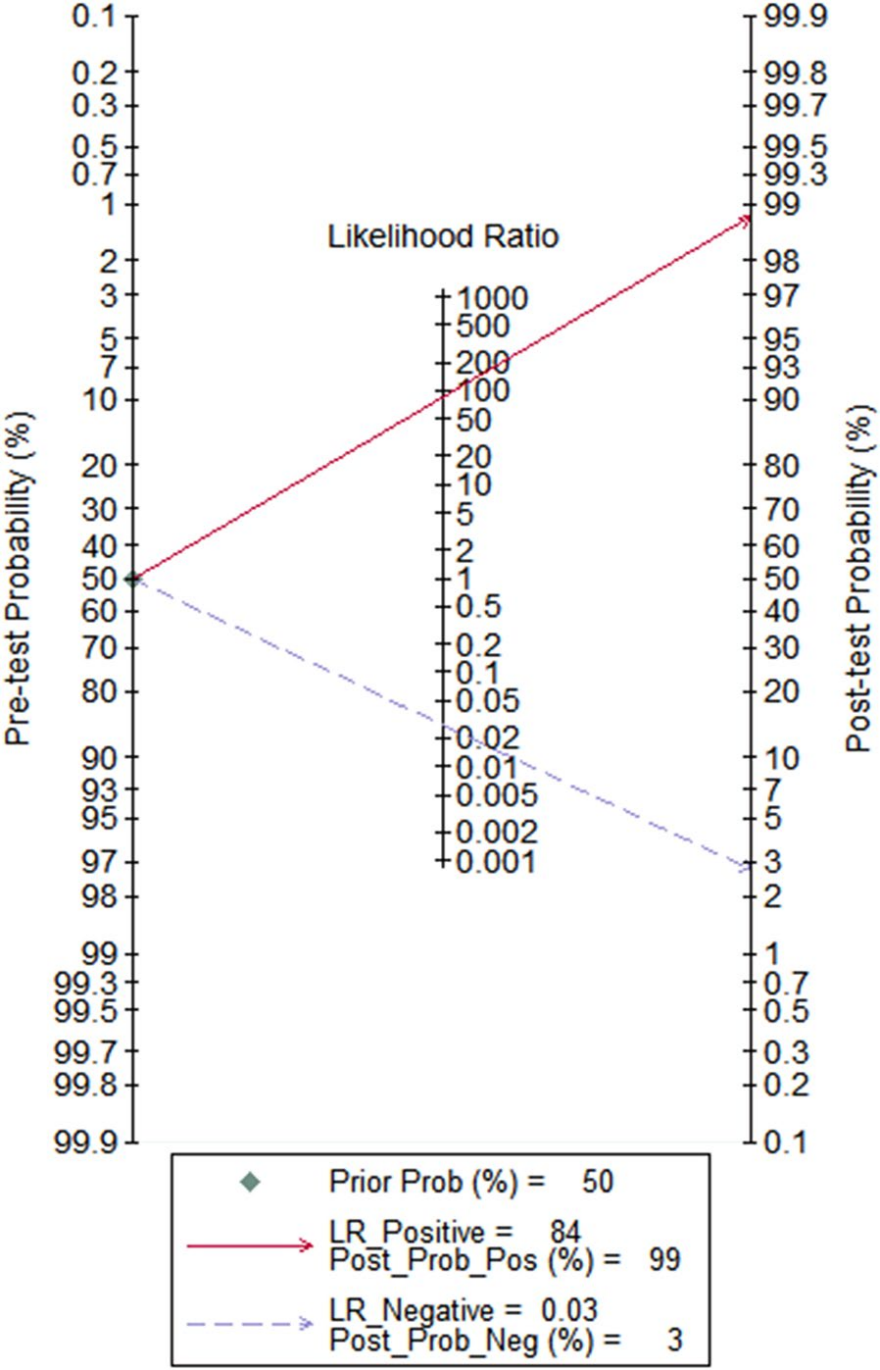
Fagan nomogram analysis of Xpert GBS LB assay

The results of sensitivity and the specificity of Xpert GBS LB were 0.96 (95% CI 0.95–0.98) and 0.94 (95% CI 0.92–0.95), as shown in Fig. 6a and b respectively. The PLR and the NLR of Xpert GBS was 15.32 (95% CI 9.20–25.53) (Fig. 6c) and 0.04 (95% CI 0.01–0.14) (Fig. 6d), respectively. The result of DOR was 1052.05 (95% CI 362.04–3057.14) (Fig. 6e). In the SROC curve of Xpert GBS LB, the AUC (Fig. 6f) was 0.9950 and the Q* index was 0.9727. The Fagan nomogram analysis showed positive post-test probability (99%) and negative post-test probability (3%) when the predicted probability was set to 50% (Fig. 7).

#### Heterogeneity analysis

**Fig. 8:**
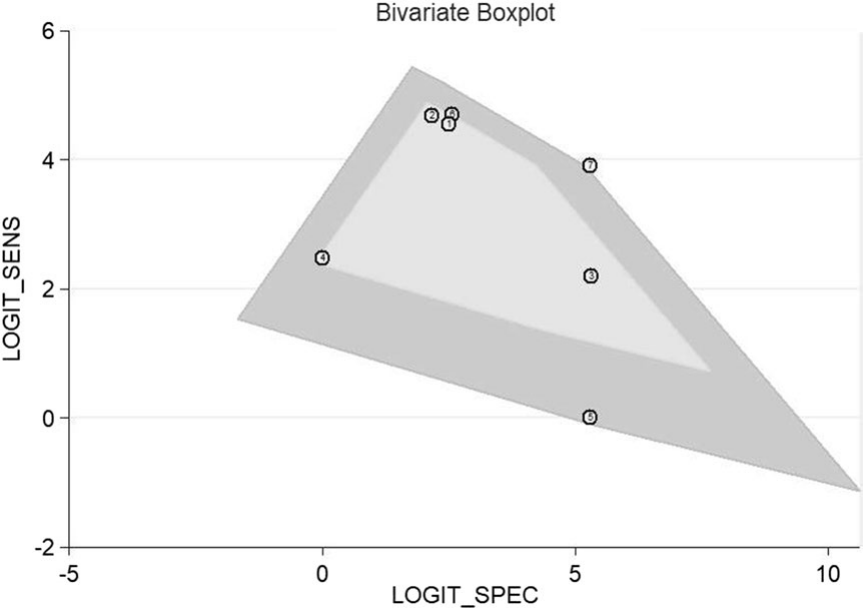
Bivariate boxplot of Xpert GBS LB assay

For the bivariate boxplot of Xpert GBS LB, one set of data out of the circles showed low heterogeneity between the included studies (Fig. 8). In addition, the I^2^ of DOR was 20.9% (Fig. 6e). The Spearman correlation coefficient of the studies about Xpert GBS LB was 0.500 and its *p* value was 0.253.

#### Publications bias evaluation

**Fig. 9:**
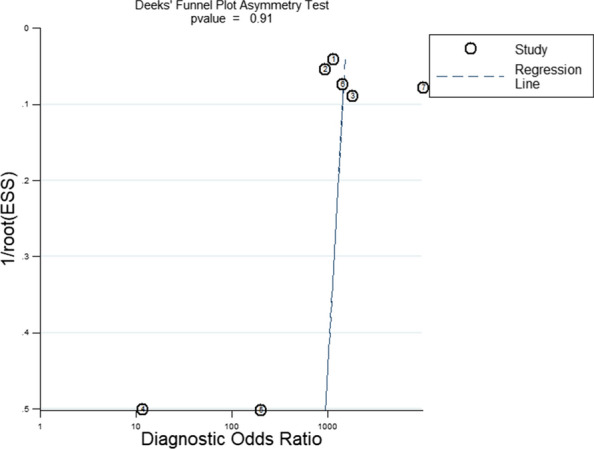
Deeks’ funnel plot asymmetry test of Xpert GBS LB assay

The Deeks’ funnel plot of Xpert GBS LB assay is shown in Fig. 9. The *p* value of Xpert GBS LB is 0.91.

## Discussion

More than 21 million pregnant women are colonized by GBS each year, which is parasitic on the vagina and/or rectum and can be vertically transmitted to the fetus during pregnancy or delivery [6, 24]. There are approximately 319,000 invasive neonatal GBS infections worldwide. Furthermore, 35% of neonatal deaths are caused by GBS infections [25]. Therefore, it is important to identify GBS infections during pregnancy and at the time of birth.

The Xpert GBS and GBS LB (Cepheid, USA) are rapid and convenient PCR assays for detecting GBS based on Cepheid’s Gene Xpert System [11]. A comprehensive search based on the inclusion and exclusion criteria set by this study led to the retrieval of 13 articles and 19 sets of data. The test methods included in these studies are Xpert GBS and GBS LB assays from Cepheid, USA, suggesting that the data we extracted would not cause great heterogeneity by different manufacturers.

The AUC (0.9806) and the Q index (0.9383) of the SROC curve for Xpert GBS are both close to 1, suggesting high diagnostic values. The results of the systematic evaluation we performed showed that Xpert GBS owned a sensitivity of 0.91 (95% CI 0.89–0.92), a specificity of 0.93 (95% CI 0.92–0.94), and a DOR of 217.19 (95% CI 62.96–749.20). Besides, the I^2^ of DOR was 92.1%. The grades of heterogeneity distinguished by I^2^ are explained as follows: 0–40% shows low heterogeneity, 50–70% shows moderate heterogeneity, and > 70% shows significant heterogeneity [26]. Further, we used a bivariate boxplot to estimate sensitivity and specificity with the corresponding 95% CI of the included studies. There are two elliptical lines in the plot, and the inner one represents the median distribution while the outer is represents 95% CI. The point out of the oval indirectly implies the threshold variability [27]. For Xpert GBS, the bivariate boxplot of Xpert GBS showed that three sets of data were out of the circles, meaning there was heterogeneity between the included studies. The result of the Spearman correlation coefficient (−0.063) was less than 0.6 and the *p* value (0.846) was greater than 0.05, indicating that there is no threshold effect in the included studies of Xpert GBS [28]. We didn’t find publication bias of Xpert GBS because the *p* value of Deeks’ funnel plot (0.73) was greater than 0.05, which indicated that the absence of asymmetry was not statistically significant [29].

For Xpert GBS LB, the AUC (0.9950) and the Q index (0.9727) of the SROC curve for Xpert GBS LB are both closer to 1, suggesting more effective diagnostic accuracy than Xpert GBS. It had a sensitivity of 0.96 (95% CI 0.95–0.98), a specificity of 0.94 (95% CI 0.92–0.95), and a DOR of 1052.05 (95% CI 362.04–3057.14). In addition, the I^2^ of DOR was 20.9%. For Xpert GBS LB, one set of data out of the circles showed low heterogeneity between included studies. Therefore, there is low heterogeneity of Xpert GBS LB. The result of the Spearman correlation coefficient (0.500) was less than 0.6 and the *p* value (0.253) was more than 0.05, showing there was no threshold effect of Xpert GBS LB assay. We didn’t find publication bias in the included studies of Xpert GBS LB because the *p* value of Deeks’ funnel plot (0.91) is greater than 0.05, indicating there was no publication bias or asymmetry in the figure.

In addition, we analyzed possible sources of heterogeneity in the inclusion studies detected by Xpert GBS: retrospective or prospective studies, differences in gestation time, delivery time, or prenatal sampling time, vaginal and rectal or amniotic fluid sampling sites, etc. A study in Lima, Peru, showed that more GBS were isolated from the vagina than the rectum. Prenatal sampling may also influence the detection of GBS [30]. Studies have shown that the colonization rate of GBS culture in the third trimester (35–37 weeks) was 29.0%, slightly lower than that in the prenatal period (29.7%) [31].

We found that the sensitivity and specificity of Xpert GBS LB assay were higher than Xpert GBS assay compared to culture. This is consistent with the results that Blake W. Buchan constructed to compare diagnosis accuracy with Xpert GBS and GBS LB assays [10]. The fundamental difference between the two methods is whether the specimen broth-enriched 18–24 h before detection. Despite the lower sensitivity and specificity, Xpert GBS assay takes less than 1 h to produce results and thus applies to intrapartum screening in delivery. Xpert GBS LB assay has higher sensitivity and specificity, but it takes more time than Xpert GBS assay, therefore applying more to antepartum screening. These two methods have their own advantages and complement each other.

However, our current research still has some limitations, as reflected in the following aspects: pregnancy time, reference standard and type of sample, which make the exhaustive evaluation of the data difficult. Furthermore, because we have not been able to contact the author to obtain the unknown timing of specimen collection, we are unable to know whether the sensitivity of Xpert GBS and GBS LB assays detected during antepartum and intrapartum is different. A comparative study in parallel of both methodologies is necessary to demonstrate the greater usefulness of one of them.

In summary, Xpert GBS and GBS LB assays have excellent accuracy in the rapid diagnosis of GBS infection in pregnant women. Therefore, further prospective studies are needed to verify whether it can be used as widely as gold standard GBS culture in clinical practice.

## Conclusions

The Xpert GBS and GBS LB assays are quick and sensitive prenatal GBS testing tools. Ideally, the test can be conducted near the delivery room to provide a fast and accurate diagnosis.

## Supplementary Information

**Additional file 1: Figure S1.** Flow diagram of study identification and inclusion.

## Data Availability

All data analyzed in this study are included in the article.

## Abbreviations

GBS: Group B *Streptococcal*
PCR: Polymerase chain reaction
DOR: Diagnosis odds ratio
AUC: Area under the curve
95% CI: Confidence interval
ACOG: American College of Obstetricians and Gynecologists
IAP: Intrapartum antibiotic prophylaxis
CDC: Centers for Disease Control and Prevention
NAAT: Nucleic acid amplification tests
QUADAS-2: Quality Assessment of Diagnostic Accuracy Studies
PLR: Positive likelihood ratio
NLR: Negative likelihood ratio
DOR: Diagnostic odds ratio
SROC: Summary receiver operating characteristic
TP: True positive
TN: True negative
Fig.: Figure
