# Supplementary figures and images for: Evaluation of Xpert GBS assay and Xpert GBS LB assay for detection of Streptococcus agalactiae
Source: Ann Clin Microbiol Antimicrob. 2021 Sep 6;20:62. doi: 10.1186/s12941-021-00461-8 (PMC8419907; doi:10.1186/s12941-021-00461-8)

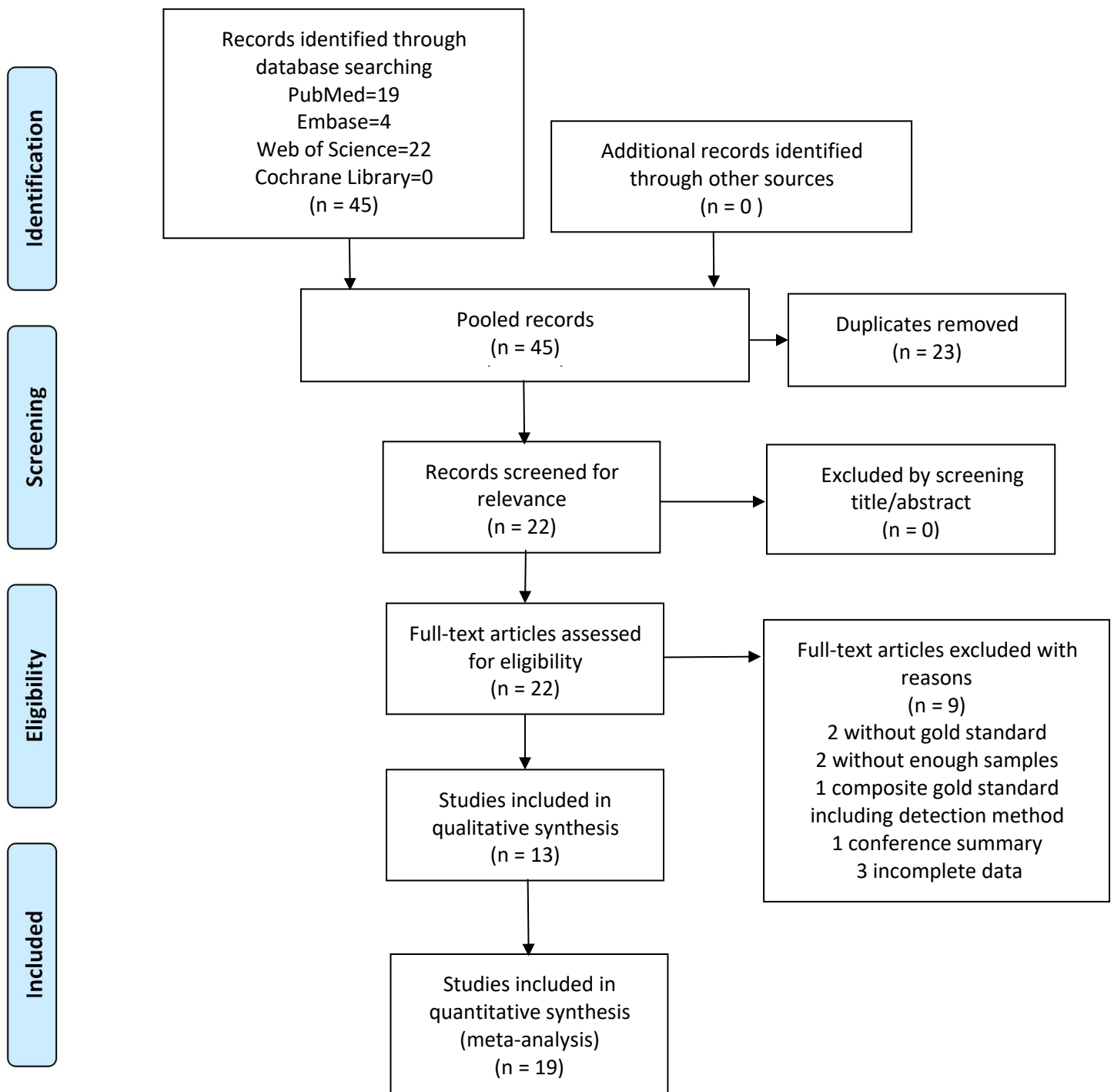

Supplement: Supplementary file 1 — Additional file 1: Figure S1. Flow diagram of study identification and inclusion. [file 12941_2021_461_MOESM1_ESM.pdf]
